# Detecting senescence: a new method for an old pigment

**DOI:** 10.1111/acel.12580

**Published:** 2017-02-09

**Authors:** Hanna Salmonowicz, João F. Passos

**Affiliations:** ^1^ Institute for Cell and Molecular Biosciences & Newcastle University Institute for AgeingNewcastle upon TyneNE4 5PLUK

**Keywords:** cellular senescence, lipofuscin, biomarker, aging

## Abstract

Cellular senescence is a state of irreversible cell cycle arrest induced by different types of cellular stresses. The field of senescence has made significant advances in the understanding of many of the mechanisms governing this phenomenon; however, a universal biomarker that unambiguously distinguishes senescent from proliferating cells has not been found. In this issue of *Aging Cell*, Evangelou and colleagues developed a sensitive method for identification of senescent cells in different types of biological material based on the detection of lipofuscin using an analogue of Sudan Black B (SBB) histochemical dye coupled with biotin, which they named GL13. The authors propose that this method is more sensitive and versatile than using SBB alone. Lipofuscin, a nondegradable oxidation product of lipids, proteins and metals, is found in senescent cells. Detection of lipofuscin using GL13 staining may be a more feasible method than others currently used for identification of senescent cells both in cell culture and tissues.

## Introduction

Cellular senescence has been defined as an irreversible cell cycle arrest which stops the propagation of damaged cells. It was first observed by Hayflick and Moorhead who demonstrated a limited replicative lifespan of human fibroblasts in culture (Hayflick & Moorhead, 1961).

Several stressors such as the shortening of telomeres, DNA lesions, oncogene activation, oxidative stress and others can induce cellular senescence (van Deursen, 2014). Depending on the trigger, senescence can be executed by several different effector pathways. The major ones comprise the p53‐p21 and p16 pathways. Senescent cells experience dramatic changes at the level of gene expression, mitochondrial function (Correia‐Melo *et al*., 2016) and epigenome (Cruickshanks *et al*., 2013). Furthermore, senescent cells have been shown to have a distinct secretome profile, known as senescence‐associated secretory phenotype (SASP) (Coppé *et al*., 2008). SASP includes growth factors, extracellular matrix degrading proteins and pro‐inflammatory cytokines. Through the SASP, senescent cells communicate with the immune system to orchestrate their own clearance and stimulate local progenitor cells to regenerate the tissue (van Deursen, 2014). However, the SASP is also capable of inducing senescence in adjacent, healthy cells, thereby contributing to tissue degeneration (Acosta *et al*., 2013). Impairment of clearance of senescent cells and chronic exposure to the SASP may result in the accumulation of senescent cells and paradoxically promote tumorigenesis.

## The challenge of identifying a universal marker of senescence

Senescent cells have been recently shown to contribute causally to the aging process. Elimination of senescent cells by suicide gene‐meditated ablation of p16^Ink4a^‐expressing senescent cells in INK‐ATTAC mice has led to improvements in healthspan and lifespan suggesting that senescent cells are drivers of aging (Baker *et al*., 2016). This has prompted the scientific community to identify new interventions to target senescence as a therapy against aging and age‐related diseases (Zhu *et al*., 2015). However, despite remarkable advances, the detection of senescent cells, particularly in tissues, is still a major challenge.

There are several reasons, both of a biological and methodological nature, which have hindered the identification of specific markers able to determine whether a cell is senescent or not:

Firstly, while senescence is characterized by numerous changes in gene expression, very few of these differences are exclusive to senescent cells. Secondly, senescence is a kinetic, multifactorial process, with several phenotypic changes occurring at different time points following the initial cell cycle arrest. This could explain why aged tissues are highly heterogeneous, possibly containing cells at different stages of the senescent programme. Thirdly, senescent cells manifest the phenotype differently depending on the type of inducing stimuli or the cell type (van Deursen, 2014). Finally, recent data have highlighted that senescence may play different physiological roles in different contexts. For instance, an ‘acute’ type of senescence has been shown to play a beneficial role during processes such as development or tissue repair (Muñoz‐Espín *et al*., 2013; Demaria *et al*., 2014), while a ‘chronic’ type of senescence may contribute to aging and age‐related disease. The recent realization that there may be different types of senescent cells in tissues has created an additional obstacle to the identification of a universal marker.

The detection of senescence‐associated β‐galactosidase (SA‐β‐Gal) activity at pH 6 is probably the most widely utilized method for identification of senescent cells (Dimri *et al*., 1995). Nevertheless, there are major limitations to this method. SA β‐Gal staining may occur in quiescent cells induced by confluency or serum starvation and in immortalized cells (Cristofalo, 2005). Furthermore, this method requires fresh, nonfixed material, which limits its applicability and the use of archived material. Its detection in tissues is technically challenging and has generated contradictory results. Given the growing realization that senescence is a multifactorial process, a multimarker approach is being favoured by many researchers in the field.

Examples of currently used markers are as follows: increased expression of cyclin kinase inhibitors p21 and p16 and absence of proliferation markers; telomere‐associated DNA damage foci (Hewitt *et al*., 2012); senescence‐associated heterochromatin foci (SAHF; Narita *et al*., 2003); loss of lamin B1 (Shimi *et al*., 2011); senescence‐associated distension of satellites (SADS) (Swanson *et al*., 2013); and expression of components of the SASP (Coppé *et al*., 2008) amongst several others. Nonetheless, there is also growing realization that many of these markers are not exclusive to all types of senescence and may only occur in specific cell types.

## Detection of lipofuscin as a senescent marker

Lipofuscin is a nondegradable aggregate of oxidized lipids, covalently cross‐linked proteins, oligosaccharides and transition metals which accumulate within lysosomes. It is a product of iron‐catalysed oxidation and polymerization reactions of a variety of cellular structures and macromolecules (Terman & Brunk, 2004). Its discovery is credited to Hannover in 1842. Multiple studies indicate that lipofuscin accumulates in various tissues and species with age, particularly postmitotic tissues such as the brain and cardiac and skeletal muscle (Terman & Brunk, 2004). However, lipofuscin has also been shown to accumulate during replicative senescence of human fibroblasts (von Zglinicki *et al*., 1995). Lipofuscin is autofluorescent and can be visualized using fluorescent microscopy; however, several other histochemical methods have been described based on lipid detection, such as staining using Sudan Black B (SBB) amongst others.

**Figure 1:**
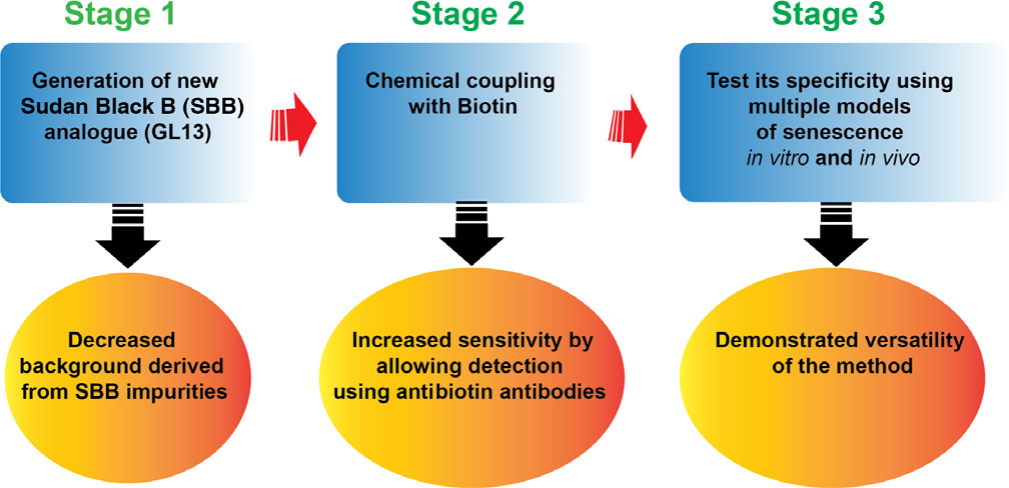
Strategy employed by Evangelou and colleagues to develop a new marker of senescence based on detection of lipofuscin.

In this article, Evangekou and colleagues designed and synthesized a structurally similar compound to SBB and coupled it to biotin. Commercially available SBB contain numerous impurities which impact on staining quality and justified the need to synthesize a new analogue. The chemical coupling with biotin allows its detection using antibiotin antibodies and thereby increases its detection sensitivity (Fig. 1). The authors show evidence for the versatility of this method: it can be detected in fresh, frozen cells and tissues, but also in fixed material. Furthermore, it can be identified in cells using both microscopy and flow cytometry. Their data indicate that GL13 staining can overcome some of the limitations of the standard SBB staining. SBB staining is less pronounced and requires a higher magnification.

The authors present multiple examples of positively stained cultured cells induced to senescence by replicative exhaustion, genotoxic stress, overexpression of p21 or oncogene activation. Furthermore, they reveal that mouse models of cellular senescence and human clinical samples of malignant lesions and irradiated tissue are characterized by elevated numbers of GL13‐positive cells, when compared to controls.

Despite its obvious advantages, users should be aware of some possible limitations of the method. For instance, lysosomes may be loaded with degradable material, described as ‘temporary lipofuscin’, but also lipid droplets and glycogen which may be detected by methods such as SBB (Terman & Brunk, 2004). It is therefore plausible that ‘false positives’ may be detected using this method.

In addition, age‐dependent accumulation of lipofuscin has been shown to occur in postmitotic tissues which in theory should not experience the phenotype originally described by Hayflick. This is at odds with the idea that lipofuscin accumulation is specific to senescent cells. One explanation is that similar pathways which drive senescence in mitotic cells can also be activated in postmitotic cells, as demonstrated in neurons (Jurk *et al*., 2012) and adipocytes (Minamino *et al*., 2009). Whether detection of lipofuscin overlaps with other senescent markers in postmitotic tissues warrants further investigation.

While the authors have convincingly demonstrated that lipofuscin accumulation correlates with senescent markers in cell culture and that lipofuscin increases in tissues with age, future work should investigate more thoroughly whether and to what extent the lipofuscin signal overlaps with other established senescent markers such as SA‐β‐Gal or p16 expression during aging *in vivo*. Finally, strategies such as the recently developed mouse models where p16‐expressing senescent cells can be specifically eliminated (Demaria *et al*., 2014; Baker *et al*., 2016) could be applied to further ascertain the specificity of this method.

A separate question which arises from this work is whether lipofuscin accumulation is a mere consequence of the induction of the senescence programme or whether its accumulation contributes causally to the development of senescence. The latter hypothesis would be consistent with early findings which demonstrated that synthetic lipofuscin could induce senescence in human fibroblasts (von Zglinicki *et al*., 1995).

The last decade has seen incredible advances in the field of cellular senescence with the realization that senescent cells play important, albeit distinct, roles *in vivo*. However, one major challenge for the community is the identification of specific markers. The novel method proposed by Evangelou and colleagues offers a possible easy and versatile solution, but whether it works as a standalone marker or should be used in combination with several others remains to be determined.

## Funding

Work in JP's laboratory is funded by the Biotechnology and Biological Sciences Research Council BBSRC.

## Conflict of interest

The authors declare no conflict of interests.
