# Corrigendum

**DOI:** 10.1111/acel.12690

**Published:** 2017-11-08

Salmonowicz H and Passos JF (2017) Detecting senescence: a new method for an old pigment. *Aging Cell* **16**, 432–434. https://doi.org/10.1111/acel.12580

In the summary of the article, ‘Detecting senescence: a new method for an old pigment’, it is mistakenly stated ‘In this issue of Aging Cell’, where it should be ‘In a previous issue of Aging Cell (Volume 16, Issue 1)’.

Furthermore, in the 2nd paragraph of the section titled ‘Detection of lipofuscin as a senescent marker’, the author's name is misspelled as ‘Evangekou’. The correct name should be ‘Evangelou’. In addition, the reference and corresponding citation should be added. The correct paragraph and additional reference are shown below:

In this article, Evangelou and colleagues designed and synthesized a structurally similar compound to SBB and coupled it to biotin (Evangelou et al., 2017). Commercially available SBB contain numerous impurities which impact on staining quality and justified the need to synthesize a new analogue. The chemical coupling with biotin allows its detection using antibiotin antibodies and thereby increases its detection sensitivity (Fig. 1). The authors show evidence for the versatility of this method: it can be detected in fresh, frozen cells and tissues, but also in fixed material. Furthermore, it can be identified in cells using both microscopy and flow cytometry. Their data indicate that GL13 staining can overcome some of the limitations of the standard SBB staining. SBB staining is less pronounced and requires a higher magnification.

Evangelou K, Lougiakis N, Rizou SV, Kotsinas A, Kletsas D, Muñoz‐Espín D, Kastrinakis NG, Pouli N, Marakos P, Townsend P, Serrano M, Bartek J, Gorgoulis VG (2017) Robust, universal biomarker assay to detect senescent cells in biological specimens. *Aging Cell*,** 16**, 192–197.

The authors would like to apologize for the inconvenience caused.

